# OLDER ADULT PERCEPTIONS OF HEALTHY AGING MEASURES

**DOI:** 10.1093/geroni/igad104.2180

**Published:** 2023-12-21

**Authors:** Payton Rule, Mathias Allemand, Patrick Hill

**Affiliations:** Washington University in St. Louis, St. Louis, Missouri, United States; University of Zurich, Zurich, Zurich, Switzerland; Washington University in St. Louis, St. Louis, Missouri, United States

## Abstract

Little is known about how lay older adults view measures commonly employed to assess healthy aging. This study examined older adults’ perceptions of scales capturing psychological wellbeing, psychosocial factors, and physical health. Participants (n = 60 US adults; MAge = 65.49, 41.18% Female, 94.12% White) were asked to rate how easy each measure was to understand and interpret on a scale of 1 (Not easy at all) to 7 (Very easy). In addition, they were asked to identify any items with which they had difficulty responding. Finally, participants provided opinions of whether these scales fully addressed their perspective on healthy aging, or if additional components were needed. Results showed that, on average, participants rated the aging-related outcomes measures as easy to understand and interpret (M = 6.14). Older adults rated measures related to physical health and health behaviors as the easiest to use (the PROMIS Health Measure (M = 6.47) and the Good Health Practices Scale (M = 6.49)), while measures that capture future planning were the most difficult to comprehend and interpret (the Consideration of Future Consequences Scale (CFC; M = 5.19) and the Goals Related to Healthy Aging Scale (M = 5.73)). While participants generally reported satisfaction with the comprehensiveness of the measures, some (22%) felt that these measures did not fully capture the social aspects of aging. Future measurement development may benefit from older adults’ input to increase their utility and better capture the experiences of older adults.

